# Optimizing panicle fertilizer application based on amylose content for balancing yield and quality of japonica rice

**DOI:** 10.3389/fpls.2026.1745001

**Published:** 2026-02-17

**Authors:** Chao Ding, Lei Xu, Zichen Wang, Liping Xu, Yongxiang Guan, Weihua Long, Longmei Wu

**Affiliations:** 1 Rural Revitalization College, Jiangsu Open University, Nanjing, Jiangsu, China; 2 Zhejiang Academy of Agricultural Sciences, Zhejiang, China; 3 Institute of Agricultural Resources and Environment, Jiangsu Academy of Agricultural Sciences, Nanjing, China; 4 Nanjing Liuhe District Agricultural Technology Extension Station, Nanjing, Jiangsu, China; 5 Jiangsu Provincial Agricultural Technology Extension Station, Nanjing, Jiangsu, China; 6 Rice Research Institute, Guangdong Academy of Agricultural Sciences, Guangzhou, Guangdong, China

**Keywords:** amylose, japonica rice, panicle nitrogen fertilizer, quality, yield

## Abstract

**Introduction:**

Panicle nitrogen application is a key agronomic practice for regulating rice yield and quality, yet its effects are highly dependent on genetic background, especially the amylose content of japonica rice varieties.

**Methods:**

This study systematically examined the differential responses to panicle nitrogen fertilizer (0, 60, 120 kg N ha^-1^) in yield formation, grain quality, and starch properties among representative japonica rice genotypes differing in amylose content (normal vs. low).

**Results and discussion:**

Results revealed variety-specific regulatory patterns: normal-amylose content (NAC) varieties showed a stronger yield response, with a 27.27% increase under the N120 treatment, largely attributable to higher panicle number per unit area. However, these varieties also exhibited significant deterioration in appearance quality, such as increased chalkiness. In contrast, low-amylose content (LAC) varieties demonstrated a greater improvement in milling quality—head rice rate increased by 10.13% under N120—but a more pronounced decline in cooking/eating quality, evidenced by reduced peak viscosity, breakdown value, and taste score. These findings highlight the need for amylose-based differential nitrogen management strategies to simultaneously achieve high yield and superior quality, providing a theoretical foundation for precision rice production oriented toward “variety-specific cultivation and quality-targeted fertilization.”

## 1 Introduction

Rice, as a staple food for nearly half of the global population, holds significant importance for national food security strategies in terms of its safe production and quality improvement. With rapid socio-economic development and a marked increase in residents’ living standards, consumer demand for rice has shifted from a singular focus on high yield to a new pattern emphasizing “high yield, high quality, and high efficiency” (Lu et al., 2025; Shao et al., 2025). In this context, how to synergistically improve rice yield and quality has become a core issue in current rice industry development.

In recent years, a series of soft japonica rice varieties with low amylose content (such as Nanjing 46, Nanjing 5055, Nanjing 9108, etc.) have been rapidly promoted due to their excellent taste (Zhang et al., 2021; Yao et al., 2024). Compared with conventional japonica rice (normal-amylose type), soft japonica rice (low-amylose type) generally exhibits poorer appearance quality, japonica rice with moderate amylose content has soft texture, good viscosity and elasticity, and is less prone to retrogradation after cooling (Zhao et al., 2019).

Panicle nitrogen fertilizer, typically applied from the young panicle differentiation stage to before heading, is a key fertilization practice for regulating rice yield formation and quality optimization. Numerous studies have shown that the application rate and timing of panicle fertilizer significantly affect rice population structure, photosynthetic capacity, and the synthesis and translocation of grain filling substances, thereby directly or indirectly determining rice yield, milling quality, appearance quality, and cooking/eating quality (Zhang et al., 2013; Zhou et al., 2023). For example, research on the high-quality taste japonica rice ‘Nanjing 9108’ showed that compared with no panicle fertilizer application, rational application of panicle fertilizer significantly increased yield by increasing the number of panicles per unit area and grains per panicle (Wang et al., 2025). However, a prominent contradiction exists in agricultural production: blindly increasing panicle fertilizer application to pursue high yield often leads to a decline in the eating quality of rice (Xiong et al., 2022). The underlying mechanism is that excessive panicle fertilizer application enhances nitrogen metabolism in the plant, relatively inhibits carbon metabolism, leading to increased protein content and decreased amylose content in the grains, which subsequently increases rice hardness, reduces viscosity and elasticity, and ultimately affects the taste value (Cao et al., 2018).

Notably, the response of carbon and nitrogen metabolism to panicle nitrogen fertilizer may differ among japonica rice varieties with different amylose contents (Hu et al., 2021; Chen et al., 2023; Wang et al., 2024; Ma et al., 2025a). Studies have shown that increasing panicle fertilizer usually decreases rice amylose content and increases protein content, but the extent of this change varies with the variety’s inherent characteristics (Tao et al., 2022; Wu et al., 2024). Furthermore, nitrogen panicle fertilizer also affects the fine structure of starch, such as altering starch granule size distribution, crystallinity, and pasting properties (e.g., peak viscosity, breakdown value), all of which are closely related to the final taste of cooked rice (Jiang et al., 2023; Shi et al., 2023; Xiong et al., 2023).

Although numerous studies have focused on the effects of nitrogen fertilizer on rice yield and quality, the majority have concentrated on single types of rice varieties (Zhu et al., 2017a; Ma et al., 2025c). Furthermore, the relationship between nitrogen application rates and the yield and quality of japonica rice with different amylose content types remains unclear, particularly the optimal nitrogen dosage for achieving a synergistic improvement in both yield and quality across these varieties. This study aims to systematically analyze the effects of different panicle nitrogen fertilizer application rates on yield components, processing quality, appearance quality, cooking/eating quality, and starch physicochemical properties using representative conventional japonica rice varieties (covering normal and low amylose content types). The expected outcomes will identify appropriate panicle fertilizer management parameters for achieving high yield and high quality in different amylose content types of japonica rice, providing a solid theoretical basis and technical support for advancing precise, high-quality rice production characterized by “cultivation according to variety and fertilization based on quality.”

## 2 Materials and methods

### 2.1 Experimental site and materials

The field experiment was conducted in 2023 at the Baima Teaching and Experimental Base of Nanjing Agricultural University (119°10′48.482″E, 31°36′51.559″N). The tested materials included four conventional japonica rice varieties with normal amylose content and four varieties with low amylose content, all widely planted in Jiangsu Province. Specific variety information is shown in **Table 1**. It was worth noting that the appearance quality of low amylose varieties is generally inferior to that of normal amylose varieties. The soil of the experimental field was sandy loam with the following basic nutrient properties: organic matter content 1.87%, total nitrogen 0.75 g/kg, available potassium 99.87 mg/kg, available phosphorus 18.48 mg/kg, ammonium nitrogen 18.55 mg/kg, alkali-hydrolyzable nitrogen 48.92 mg/kg, pH 6.93.

**Table 1:** The information on the japonica rice cultivars with different amylose contents.

Type	Variety	Amylose content (%)	Year of offical release	Breeding organization
NAC	Sidao 301	16.7	2017	Suqian Institute of Agricultural Sciences, Jiangsu Academy of Agricultural Sciences
	Ninggeng 7	15.6	2011	Nanjing Agricultural University
	Suxiu 867	16.3	2010	Jiaxing Academy of Agricultural Sciences
	Huaidao 5	16.5	2000	Huaiyin Agricultural Science Research Institute in Xuzhou-Huai’an Region, Jiangsu
LAC	Nangeng 5718	12.1	2015	Institute of Food Crops, Jiangsu Academy of Agricultural Sciences
	Taixianggeng 1402	10.7	2018	Jiangsu Hongqi Farm Ecological Agriculture Co., Ltd.
	Nanjing 9108	12.5	2009	Institute of Food Crops, Jiangsu Academy of Agricultural Sciences
	Jingxiangyu1	11.7	2017	Lixiahe Regional Agricultural Science Research Institute, Jiangsu

NAC, japonica rice with normal amylose content; LAC, japonica rice with low amylose content.

### 2.2 Experimental design and cultivation management

The experiment used a split-plot design. Nitrogen fertilizer treatment was the main plot, with three nitrogen panicle fertilizer levels (0, 60, 120 kg N ha^-1^). Panicle fertilizer was applied at a 1:1 ratio during the fourth leaf stage from the top (promoting flower differentiation) and the second leaf stage from the top (protecting flower development), which is a commonly used period for panicle fertilizer application. Variety was the subplot. There were three replications, totaling 9 main plots and 72 subplots. Each subplot area was 30 m². Seedlings were raised using dry nursery methods. Sowing date was May 24; transplanting date was June 17. Transplanting density was 30.0×12 cm with multiple seedlings per hill. The basal and tillering fertilizer application rate was 180 kg N ha^-1^ pure nitrogen. Phosphorus fertilizer (P_2_O_5_) application rate was 90 kg ha^-1^, all applied as basal fertilizer. Potassium fertilizer (K_2_O) application rate was 120 kg ha^-1^, applied equally as basal fertilizer and before jointing (promoting spikelet fertilizer). Water management was strictly carried out according to high-yield field requirements. Diseases and pests were controlled promptly.

### 2.3 Measurements and methods

#### 2.3.1 Yield and yield component measurements

At maturity, a small combine harvester was used for yield measurement after removing border rows. The harvested area per plot was 9 m². Grains were air-dried and weighed. Grain moisture content was measured using a grain moisture meter (PM-8818-A, Kett Electric Laboratory, Tokyo, Japan) (average of three measurements per sample). Yield was then converted to a moisture content of 14.5%. Finally, yield per unit area was calculated based on the actual harvested area. The number of panicles per unit area was calculated from a census of 50 hills per plot (5 rows×10 hills). Fifteen single panicles were taken from each plot to determine seed setting rate and 1000-grain weight. Grains per panicle were calculated based on actual yield, panicle number, seed setting rate, and 1000-grain weight.

#### 2.3.2 Measurement of major rice quality indicators

Rice was harvested at maturity, threshed, and air-dried to standard moisture content. After storage for three months to stabilize physicochemical properties, three 150g samples were taken from each treatment. The analysis of rice quality followed the Chinese National Standard GB/T 17891–2017 (GB/T 17891-2017, 2017). The milling quality, including brown rice percentage, milled rice percentage, and head rice percentage, was assessed gravimetrically. Simultaneously, the appearance quality parameters, such as grain length, grainwidth, chalky grain rate, and chalkiness degree, were determined using a rice appearance quality analyzer (SC-E, Hangzhou Wanshen Test Technology Co., Ltd., China).

Taste value was measured using a rice taste meter (STSRFN1A, Satake, Japan). A random 30g sample of milled rice was used to determine total protein content using a near-infrared rapid quality analyzer (Infratec 1241 Grain Analyzer, Foss Tecator, Sweden). Amylose content determination: Amylose content was determined using the iodine blue colorimetric method. Absorbance of standard samples with known amylose content was measured at 720 nm wavelength using a 722N visible spectrophotometer (Shanghai Yidian Analysis Instrument Co., Ltd.). A standard curve was plotted with absorbance as the x-axis and amylose content as the y-axis. The amylose content of the test samples was determined from the standard curve based on their absorbance.

Rice flour RVA profile characteristics: Determined using a Super 3 Rapid Visco Analyzer (RVA) (Newport Scientific Instruments, Australia) according to the AACC (American Association of Cereal Chemists) standard method (1995-61-02) RICE. Data were analyzed using the TWC (Thermal Cycle for Windows) software.

### 2.4 Data calculation and statistical analysis

Data processing was performed using Microsoft Excel 2016. Analysis of variance (ANOVA) was conducted using IBM SPSS Statistics 25 to analyze the effects of fertilization practices and variety type, and their interactions, on the measured indicators (at the 0.05 level). The Least Significant Difference (LSD) method was used to compare the significance of differences between means (at the 0.05 level). All figures presented in this study were created with OriginPro 2024b (OriginLab Corporation, USA).

## 3 Results

### 3.1 Yield and yield components​

**Figure 1:**
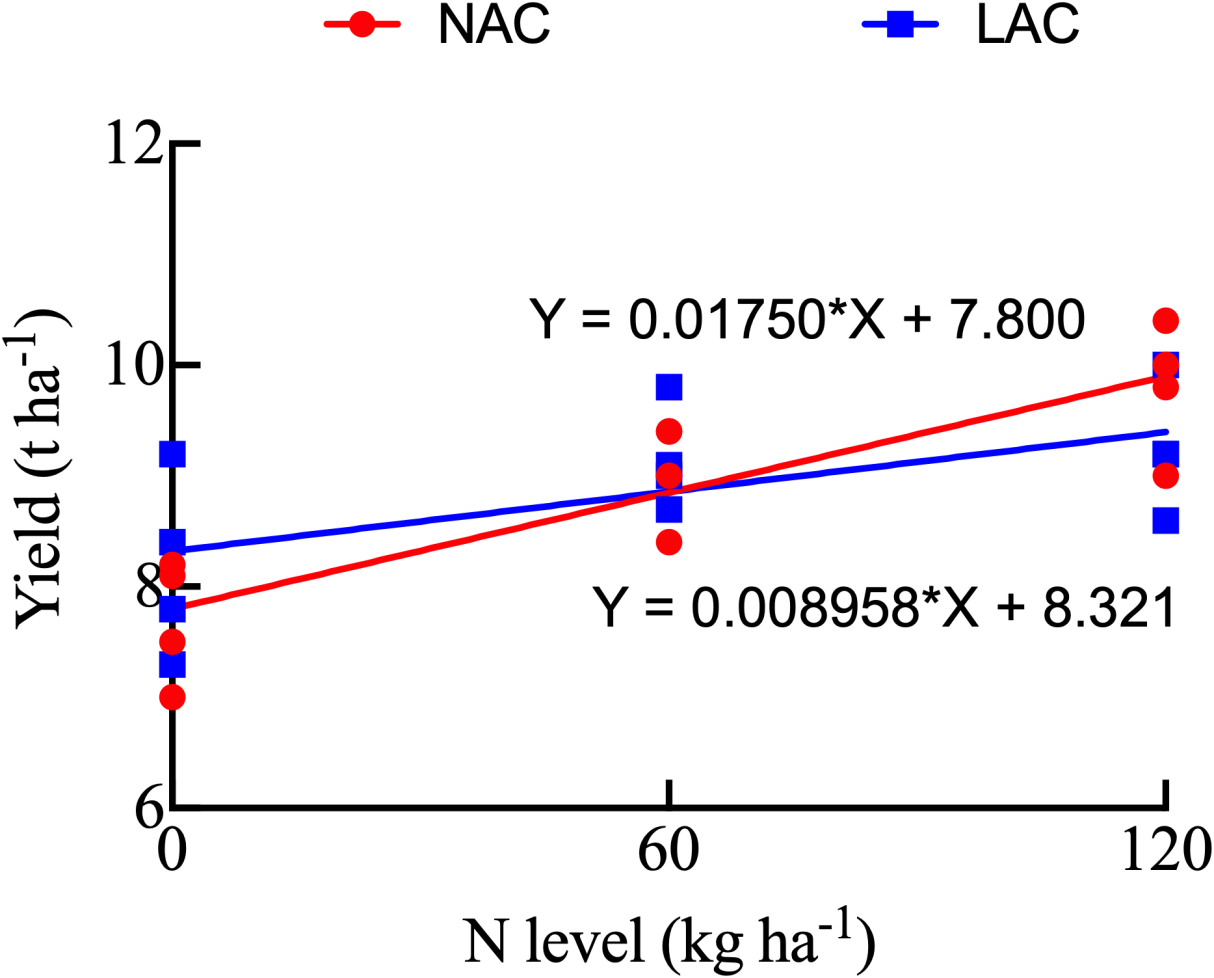
Relationship between panicle nitrogen level and yield of japonica rice. NAC, japonica rice with normal amylose content; LAC, japonica rice with low amylose content.

As the application rate of panicle nitrogen fertilizer increased, the yield of NAC varieties showed a continuous upward trend (**Table 2**). The average yield of NAC varieties under the N0 treatment was 7.70 *t* ha^-1^. Compared to N0, the yield increase under N60 was 17.53%, and it was 27.27% under N120. The yield of LAC varieties also increased with increasing nitrogen application level, but the increase was relatively smaller conpared to NAC varieties. The average yield of LAC varieties under N0 was 8.18 *t* ha^-1^. Compared to N0, the yield increase under N60 was 11.93%, and under N120 it was 13.15%. General Linear Equation was established based on the yield and nitrogen level (**Figure 1**). According to the linear equation, compared with LAC varieties, NAC varieties can achieve higher yields under high nitrogen levels, demonstrating greater yield potential. Both types showed a significant increase in panicle number and total spikelets with increasing panicle nitrogen fertilizer, The NAC varieties had a larger increase than the LAC varieties. The seed setting rate of both types decreased with increasing nitrogen application. Nitrogen treatment had no significant effect on grain weight and spikelets per panicle.

**Table 2:** Yield and yield components of the japonica rice with different amylose contents under different panicle nitrogen fertilizer.

T	V	N	Panicle number (×10^4^ ha^-1^)	Spikelets per panicle	Total spikelets (×10^8^ ha^-1^)	Seed setting rate (%)	Grain weight (mg)	Yield (t ha^-1^)
NAC	S301	N0	302.9 b	123.2 b	3.73 c	91.0 a	24.2 a	8.2 c
		N60	309.6 b	139.2 a	4.31 b	89.1 ab	24.5 a	9.4 b
		N120	344.7 a	141.1 a	4.86 a	85.6 b	24.9 a	10.4 a
	N7	N0	170.2 c	182.7 b	3.11 c	87.5 a	25.7 a	7.0 c
		N60	265.8 b	186.5 ab	4.96 b	74.2 b	24.5 b	9.0 b
		N120	289.4 a	198.2 a	5.74 a	69.9 c	24.5 b	9.8 a
	S867	N0	223.2 c	158.4 a	3.54 c	89.7 a	25.4 a	8.1 c
		N60	263.6 b	168.1 a	4.43 b	88.0 a	24.1 b	9.4 b
		N120	327.5 a	156.6 a	5.13 a	82.5 b	23.7 b	10.0 a
	H5	N0	261.8 c	128.2 a	3.36 c	90.3 a	24.9 a	7.5 c
		N60	303.4 b	126.6 a	3.84 b	88.5 a	24.7 a	8.4 b
		N120	324.3 a	134.3 a	4.36 a	83.8 b	24.7 a	9.0 a
LAC	N5718	N0	260.6 c	157.3 a	4.10 c	88.7 a	23.2 a	8.4 b
		N60	301.5 b	142.2 b	4.29 b	90.1 a	23.3 a	9.0 a
		N120	367.0 a	127.5 c	4.68 a	87.3 a	22.6 a	9.2 a
	T1402	N0	281.5 c	150.2 b	4.23 b	84.7 a	25.7 a	9.2 b
		N60	367.7 a	140.4 b	5.16 ab	77.0 b	24.7 b	9.8 ab
		N120	316.2 b	172.3 a	5.45 a	74.1 c	24.9 b	10.0 a
	N9108	N0	274.8 b	133.0 b	3.65 c	87.1 a	23.0 a	7.3 b
		N60	285.0 b	153.9 a	4.39 b	87.1 a	22.8 a	8.7 a
		N120	356.2 a	140.1 ab	4.99 a	76.6 b	22.5 a	8.6 a
	J1	N0	217.2 b	193.0 a	4.19 b	77.4 a	24.0 a	7.8 b
		N60	267.8 a	206.5 a	5.53 a	68.9 b	23.9 a	9.1 a
		N120	281.6 a	198.4 a	5.59 a	67.7 b	24.3 a	9.2 a
ANOVA		T	ns	ns	ns	ns	ns	ns
		N	**	ns	**	*	ns	**
		T×N	*	ns	*	ns	ns	**

T, type; V, variety; N, panicle nitrogen treatments; N0, 0 kg N ha^-1^; N60, 60 kg N ha^-1^; N120, 120 kg N ha^-1^; NAC, japonica rice with normal amylose content; LAC, japonica rice with low amylose content; Different lowercase letters indicate significant differences among treatments in the same variety at P<0.05. ns, no significance. *, significant difference at 0.05 probability level. **, significant difference at 0.01 probability level.

### 3.2 Appearance quality

LAC varieties responded more noticeably to nitrogen treatment, with grain length increasing by 4.72% under N60. Grain width of LAC varieties gradually increased with nitrogen application, while it decreased in NAC varieties. The chalky grain rate increased with nitrogen application in both types, with the NAC varieties showing the largest increase of 20.90% under N120. NAC varieties were more sensitive to nitrogen treatment regarding chalkiness degree, which increased by 23.62% under N120 (**Table 3**).

**Table 3:** Appearance quality of japonica rice cultivars with different amylose contents under different panicle nitrogen fertilizer.

T	V	N	GL (mm)	GW (mm)	L/W	CR (%)	CD (%)
NAC	S301	N0	4.80 b	2.60 a	1.84 b	17.5 c	5.1 c
		N60	4.85 b	2.61 a	1.86 b	29.2 b	9.2 b
		N120	4.99 a	2.56 a	1.94 a	34.2 a	11.6 a
	N7	N0	4.29 b	2.59 a	1.65 b	31.1 b	11.8 b
		N60	4.49 a	2.61 a	1.71 a	32.0 b	12.5 ab
		N120	4.39 ab	2.54 b	1.73 a	36.0 a	13.8 a
	S867	N0	4.44 ab	2.56 ab	1.73 a	15.5 c	5.6 b
		N60	4.26 b	2.48 b	1.71 a	25.8 b	8.8 ab
		N120	4.53 a	2.60 a	1.75 a	32.8 a	10.3 a
	H5	N0	4.40 b	2.69 a	1.63 b	49.2 c	17.6 c
		N60	4.56 a	2.70 a	1.69 a	62.2 b	21.9 b
		N120	4.42 b	2.63 b	1.68 a	69.1 a	24.4 a
LAC	N5718	N0	3.75 a	2.63 a	1.42 b	53.6 b	19.4 b
		N60	3.50 b	2.40 b	1.51 a	61.1 a	25.1 a
		N120	3.67 ab	2.54 ab	1.44 b	58.9 ab	23.3 a
	T1402	N0	4.11 b	2.56 b	1.60 a	69.7 a	30.3 a
		N60	4.34 a	2.66 a	1.63 a	70.4 a	29.1 a
		N120	4.18 b	2.61 ab	1.61 a	65.7 b	24.6 b
	N9108	N0	3.70 b	2.43 b	1.52 b	67.5 b	23.3 c
		N60	4.13 a	2.62 a	1.57 a	73.9 a	28.1 a
		N120	3.91 b	2.54 ab	1.53 b	67.3 b	25.9 b
	J1	N0	3.69 b	2.38 b	1.55 b	65.1 a	25.1 a
		N60	3.99 a	2.54 a	1.56 b	63.0 ab	27.2 a
		N120	4.04 a	2.48 ab	1.64 a	61.4 b	26.7 a
ANOVA		T	**	ns	**	**	**
		N	ns	ns	ns	ns	ns
		T×N	ns	ns	ns	*	**

T, type; V, variety; N, panicle nitrogen treatments; N0, 0 kg N ha^-1^; N60, 60 kg N ha^-1^; N120, 120 kg N ha^-1^; NAC, japonica rice with normal amylose content; LAC, japonica rice with low amylose content; GL, grain length; GW, grain width; L/W, length/width; CR, chalkiness rate; CD, chalkiness degree. Different lowercase letters indicate significant differences among treatments in the same variety at P<0.05. ns, no significance. *, significant difference at 0.05 probability level. **, significant difference at 0.01 probability level.

### 3.3 Milling quality

The milling quality of NAC varieties was significantly better than that of LAC varieties (**Table 4**). LAC varieties responded more positively to nitrogen panicle fertilizer treatment, with all indicators improving with increasing nitrogen. The response of NAC varieties was relatively conservative, with some indicators even slightly decreasing. With increasing nitrogen panicle fertilizer, the brown rice rate of low amylose varieties increased significantly (3.56% higher in N120 than N0), while NAC varieties showed no significant change. The milled rice rate of LAC varieties increased with nitrogen panicle fertilizer (3.65% higher in N120 than N0), while it slightly decreased in NAC varieties (1.76% lower). The head rice rate of LAC varieties increased significantly with nitrogen (10.13% higher in N120 than N0), while it slightly decreased in NAC varieties (2.21% lower).

**Table 4:** Milling quality of japonica rice cultivars with different amylose contents under different panicle nitrogen fertilizer.

T	V	N	BR (%)	MR (%)	HR (%)
NAC	S301	N0	81.9 ab	73.1 a	58.9 a
		N60	83.2 a	70.3 b	57.6 b
		N120	80.8 b	69.6 b	56.1 c
	N7	N0	81.7 a	71.8 a	58.2 a
		N60	80.9 a	69.7 a	56.1 b
		N120	80.5 a	71.9 a	54.4 c
	S867	N0	80.4 b	71.4 a	62.4 a
		N60	81.3 ab	70.1 a	60.3 b
		N120	82.3 a	71.1 a	59.6 b
	H5	N0	83.4 b	74.8 a	58.5 a
		N60	83.9 b	72.7 b	58.0 a
		N120	85.8 a	73.3 b	55.7 b
LAC	N5718	N0	83.1 a	69.3 a	33.6 b
		N60	83.5 a	69.7 a	33.8 b
		N120	83.7 a	69.6 a	36.9 a
	T1402	N0	78.0 b	66.1 b	50.8 b
		N60	83.7 a	71.8 a	51.5 ab
		N120	84.3 a	71.4 a	52.0 a
	N9108	N0	82.1 b	71.4 b	47.2 b
		N60	83.3 ab	72.3 ab	53.4 ab
		N120	84.1 a	73.4 a	54.2 a
	J1	N0	79.1 c	65.1 b	41.6 c
		N60	80.9 b	68.1 a	48.2 b
		N120	82.7 a	68.7 a	51.4 a
ANOVA		T	ns	*	**
		N	ns	ns	ns
		T×N	ns	ns	*

T, type; V, variety; N, panicle nitrogen treatments; N0, 0 kg N ha^-1^; N60, 60 kg N ha^-1^; N120, 120 kg N ha^-1^; NAC, japonica rice with normal amylose content; LAC, japonica rice with low amylose content; BR, brown rice rate; MR, milled rice rate; HD, head rice rate. Different lowercase letters indicate significant differences among treatments in the same variety at P<0.05. ns, no significance. *, significant difference at 0.05 probability level. **, significant difference at 0.01 probability level.

### 3.4 Eating quality and nutritional quality

The taste value decreased with increasing panicle nitrogen fertilizer for both variety types (**Table 5**). Compared to N0, the taste value of LAC varieties under N120 decreased by 21.75%, while the decrease was relatively smaller for NAC varieties (14.29%). The protein content increased with increasing panicle nitrogen fertilizer for both variety types. Compared to N0, the protein content of NAC varieties under N60 and N120 increased by 33.56% and 65.37%, respectively. The protein content of LAC varieties exhibited an increase of comparable magnitude.

**Table 5:** Rice taste values and nutritional qualities of japonica rice cultivars with different amylose contents under different panicle nitrogen fertilizer.

T	V	N	Taste value	Protein content (%)
NAC	S301	N0	89.5 a	6.6 c
		N60	82.0 b	9.2 b
		N120	74.5 c	11.0 a
	N7	N0	90.0 a	6.4 c
		N60	80.5 b	9.2 b
		N120	70.0 c	11.6 a
	S867	N0	87.5 a	7.6 c
		N60	81.0 a	9.2 b
		N120	75.0 b	10.4 a
	H5	N0	86.5 a	7.7 c
		N60	78.5 b	10.2 b
		N120	71.5 c	13.8 a
LAC	N5718	N0	89.0 a	8.0 c
		N60	78.0 b	11.1 b
		N120	71.5 c	12.9 a
	T1402	N0	87.0 a	8.2 c
		N60	77.5 b	10.5 b
		N120	69.0 c	12.3 a
	N9108	N0	88.5 a	7.8 c
		N60	77.5 b	10.7 b
		N120	67.5 c	13.4 a
	J1	N0	89.5 a	7.9 c
		N60	79.0 b	11.2 b
		N120	69.0 c	13.1 a
ANOVA		T	ns	*
		N	**	**
		T×N	**	*

T, type; V, variety; N, nitrgen panicle treatments; N0, 0 kg N ha^-1^; N60, 60 kg N ha^-1^; N120, 120 kg N ha^-1^; NAC, japonica rice with normal amylose content; LAC, japonica rice with low amylose content. Different lowercase letters indicate significant differences among treatments in the same variety at P<0.05. ns, no significance. *, significant difference at 0.05 probability level. **, significant difference at 0.01 probability level.

### 3.5 Starch pasting properties

Peak viscosity, hot paste viscosity, final viscosity, and breakdown value decreased with increasing nitrogen panicle fertilizer for both types, with the decrease being more pronounced in LAC varieties (**Table 6**). Compared to N0, under N120, LAC varieties showed decreases of 18.37% in peak viscosity, 19.73% in hot paste viscosity, and 13.02% in final viscosity. NAC varieties under N120 showed decreases of 12.85% in peak viscosity, 18.83% in hot paste viscosity, and 10.50% in final viscosity. The setback value of LAC varieties increased with increasing nitrogen panicle fertilizer, while changes in NAC varieties were small.

**Table 6:** Rapid visco-analyzer (RVA) profile characteristics of japonica rice cultivars with different amylose contents under different panicle nitrogen fertilizer.

T	V	N	PV (cP)	TV (cP)	FV (cP)	BD (cP)	SB (cP)
NAC	S301	N0	2963.5 a	1847.5 b	2910.0 a	1116.0 a	-53.5 a
		N60	3134.0 a	2050.0 a	3096.0 a	1084.0 a	-38.0 a
		N120	2791.5 b	1769.0 b	2825.0 b	1022.5 a	33.5 a
	N7	N0	3151.5 a	2287.0 a	3307.0 a	864.5 a	155.5 b
		N60	2872.5 b	2037.5 b	3105.5 b	835.0 a	233.0 a
		N120	2525.5 c	1557.5 c	2774.0 c	968.0 a	248.5 a
	S867	N0	3379.0 a	2239.5 a	3193.0 a	1139.5 a	-186.0 b
		N60	3172.5 b	2142.5 a	3115.5 a	1030.0 a	-57.0 a
		N120	2851.0 c	1827.5 b	2802.5 b	1023.5 a	-48.5 a
	H5	N0	3393.5 a	2296.5 a	3299.0 a	1097.0 b	-94.5 a
		N60	3245.5 a	2177.5 a	3168.5 a	1068.0 b	-77.0 a
		N120	3063.5 b	1884.0 b	2973.0 b	1179.5 a	-90.5 a
LAC	N5718	N0	3839.5 a	2109.5 a	2718.0 a	1730.0 a	-1121.5 c
		N60	3292.0 b	1722.5 b	2310.5 b	1569.5 b	-981.5 b
		N120	2657.0 c	1386.0 c	1912.0 c	1271.0 c	-745.0 a
	T1402	N0	3528.5 a	1959.5 a	2570.0 b	1569.0 a	-958.5 b
		N60	3463.0 a	2089.0 a	2914.5 a	1374.0 b	-548.5 a
		N120	2910.5 b	1701.5 b	2290.5 c	1209.0 c	-620.0 a
	N9108	N0	2941.5 a	1759.0 a	2404.5 a	1182.5 a	-537.0 b
		N60	2721.0 b	1559.5 b	2172.5 b	1161.5 ab	-548.5 b
		N120	2739.0 b	1595.0 b	2361.5 ab	1144.0 b	-377.5 a
	J1	N0	3182.5 a	2045.0 a	2688.5 a	1137.5 ab	-494.0 ab
		N60	2881.5 b	1653.5 b	2305.0 b	1228.0 a	-576.5 b
		N120	2707.5 b	1637.0 b	2465.5 b	1070.5 b	-242.0 a
ANOVA		T	ns	*	**	**	**
		N	*	**	*	ns	ns
		T×N	ns	ns	ns	*	ns

T, type; V, variety; N, nitrgen panicle treatments; N0, 0 kg N ha^-1^; N60, 60 kg N ha^-1^; N120, 120 kg N ha^-1^; NAC, japonica rice with normal amylose content; LAC, japonica rice with low amylose content; PV, peak viscosity; HV, hot viscosity; FV, final viscosity; BD, breakdown; SB, setback. Different uppercase letters indicate significant differences among treatments in the same variety at P<0.05. ns, no significance. *, significant difference at 0.05 probability level. **, significant difference at 0.01 probability level.

### 3.6 Relationships among rice yield and quality traits

**Figure 2:**
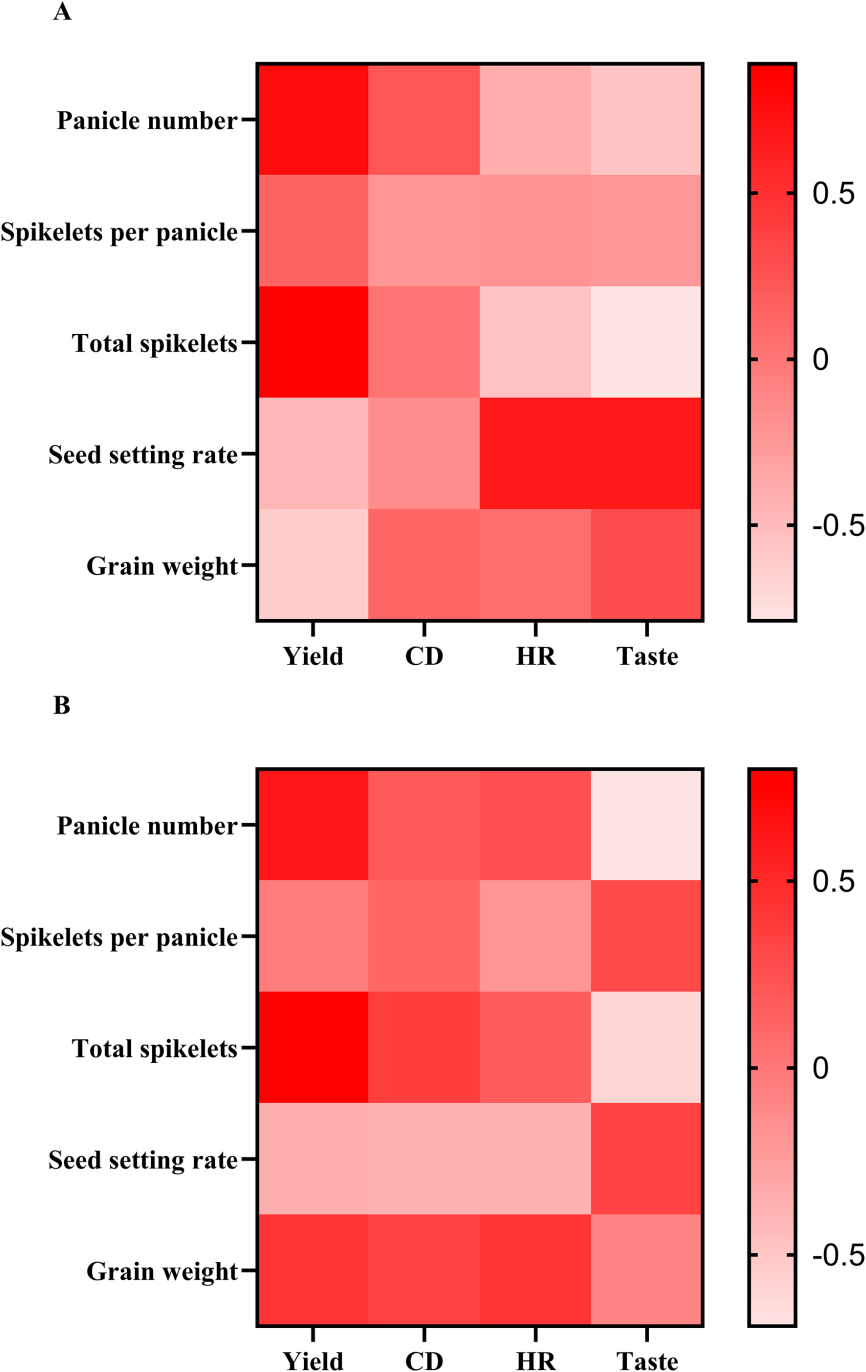
Correlation between the yield components and yield, CD, HD, taste in NAC varieties **(A)** and LAC varieties **(B)**. CD, chalkiness degree; HD, head rice rate.

**Figure 3:**
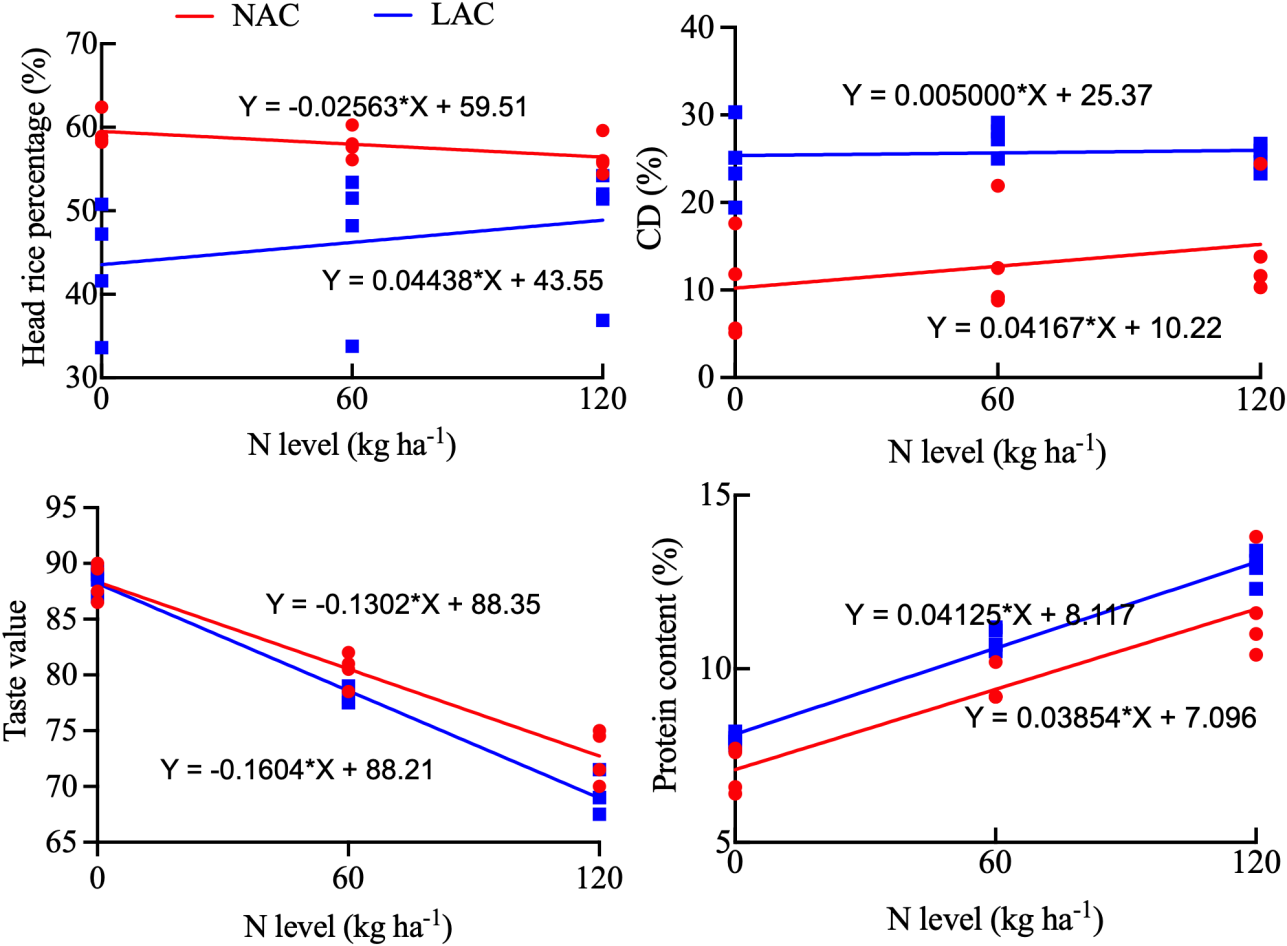
Relationship between panicle nitrogen level and grain quality of japonica rice.

Correlation analysis indicated that in NAC varieties, panicle number and total spikelets showed high correlation coefficients with yield, while the seed setting rate exhibited high correlation coefficients with head rice rate and taste value. Similarly, grain weight demonstrated high correlation coefficients with chalkiness degree, head rice rate and taste value. In LAC varieties, panicle number and total spikelets also showed high correlation coefficients with yield. Meanwhile, spikelets per panicle and seed setting rate were highly correlated with taste value. Grain weight had relatively high correlation coefficients with yield, chalkiness degree and head rice rate (**Figure 2**). Relationship between rice quality traits and nitrogen level was shown in **Figure 3**. Compared with LAC varieties, NAC varieties exhibited a smaller reduction in taste value with increasing nitrogen fertilization. While the protein content of both types increased in response to higher nitrogen levels, the extent of increase was essentially comparable.

## 4 Discussion

### 4.1 The mechanism of nitrogen panicle fertilizer regulates the yield of rice varieties with different amylose contents

The core finding of this study is that there are fundamental differences in the response patterns to panicle nitrogen fertilizer among japonica rice varieties with different amylose contents. Specifically, compared to LAC varieties, NAC varieties have greater potential for yield increase after applying additional panicle fertilizer, mainly due to a significant increase in total spikelets per unit area and no significant reduction in grain weight. This highlights the importance of applying panicle fertilizer in securing tiller development into effective panicles and promoting grain filling. Sufficient nitrogen supply can prolong the functional period of leaves, providing more photosynthetic products to fill a larger sink capacity, thereby maximizing yield potential, especially for NAC varieties. This is consistent with the results of Jiang (2023) and Liu (2022), who suggested that the increase in total spikelets per unit area is the main reason for yield increase from nitrogen panicle fertilizer application (Liu et al., 2022; Jiang et al., 2023).

The fundamental reason for the observed differences in yield response to nitrogen panicle fertilizer among the NAC varieties and LAC varieties likely lies in their distinct intrinsic carbon-nitrogen (C-N) metabolic balance and their corresponding response strategies to nitrogen supply. High-amylose varieties generally possess a stronger capacity to direct carbon metabolism towards starch synthesis. Ma (2025b) indicates that the activity of granule-bound starch synthase (GBSS) in the grains of high-amylose japonicarice during the grain-filling period is higher than in low-amylose varieties (Ma et al., 2025b). Furthermore, high-amylose rice varieties are characterized by higher chlorophyll content in leaves, elevated levels of soluble sugars in leaves and stem sheaths, and a longer duration of soluble sugar supply from the stem sheaths (Jie, 2004). These traits provide a more abundant substrate for amylose synthesis. When nitrogen application promotes nitrogen metabolism (protein synthesis), a robust carbon metabolism foundation can better support C-N balance, preventing carbohydrate deficiency due to excessive nitrogen, thereby enabling a more substantial yield increase.

### 4.2 The mechanism of nitrogen panicle fertilizer regulates the qualities of rice varieties with different amylose contents

The appearance quality (e.g., chalkiness degree) of NAC varieties was more sensitive to high nitrogen levels, showing a significant deterioration trend. In contrast, the processing quality (e.g., brown rice rate, milled rice rate, head rice rate) of LAC varieties showed a more positive response to increased panicle fertilizer. Notably, although the cooking/eating quality of both types decreased with increasing panicle fertilizer, the decrease in taste value and related pasting properties (e.g., peak viscosity, breakdown value) was more significant in LAC varieties.

The underlying reason for the varietal differences in quality response to nitrogen fertilizer observed in this study cannot be attributed to changes in protein or amylose content, as protein content responded similarly across varieties and nitrogen fertilizer showed no significant effect on amylose content. The initial hypothesis is that the differences in quality response to nitrogen may stem from inherent variations in carbon-nitrogen (C-N) metabolism between the varieties. The stronger carbon metabolism *“*substrate*”* in High-amylose varieties might partially buffer the adverse effects of excessive nitrogen metabolism on starch synthesis (Xia et al., 2022). This could explain their relatively smaller deterioration in eating quality. Conversely, the intrinsic carbon metabolism in Low-amylose varieties is relatively weaker, and their superior eating quality relies on a delicate allocation of carbon flux. High nitrogen levels significantly enhance their nitrogen metabolism, disrupting the inherent C-N balance (Xi et al., 2020; Guo et al., 2022). This leads to a greater proportion of photosynthetic products being diverted towards the synthesis of nitrogenous compounds like proteins, thereby relatively inhibiting starch synthesis and accumulation. This manifests directly as a significant decrease in peak viscosity and breakdown value, resulting in poorer eating quality.

Another potential reason may be related to differences in how the proportion of short chains in amylopectin responds to nitrogen fertilizer between japonicarice varieties with different amylose contents.​ Zhu (2017b) found that as nitrogen levels increased, starch granule size decreased, the granule surfaces became more uneven with more pits, the relative crystallinity of starch increased, and the short-range molecular order of starch decreased (Zhu et al., 2017b). Zhou (2020) discovered that increased nitrogen application reduced the amylose-to-amylopectin ratio and increased the proportion of short chains in amylopectin (Zhou et al., 2020). Huang (2020) reported that under nitrogen treatment, the proportion of short amylopectin chains decreased in the low-amylose japonicavariety Nanjing 9108, but increased in the conventional-amylose japonicavariety Huaidao 5. This divergence is regulated by the expression of genes related to amylopectin synthesis, such as SSIand BEIIb (Huang et al., 2020). Currently, there is a lack of systematic comparative studies on the response of starch fine structure in grains of varieties with different amylose contents to nitrogen fertilizer. This gap warrants further investigation and validation in future research.

This study emphasizes the necessity of incorporating varietal genetic background (e.g., amylose content) as a key factor into nitrogen management theoretical models. It shows that the synergy between high yield and high quality is not an absolute contradiction, but the path to achieving it highly depends on the metabolic type of the variety. Future research could further investigate the mechanisms behind the differential responses of yield and quality to nitrogen fertilizer in varieties with varying amylose contents from the perspective of carbon−nitrogen metabolic interactions. The research results have direct guiding significance for high-quality and high-yield rice cultivation. For high amylose varieties, yield potential can be explored by moderately increasing panicle fertilizer, while monitoring the risk of appearance quality decline. For low amylose type high-quality soft rice varieties, the primary principle for quality production is to avoid excessive panicle fertilizer application. The fertilization strategy should be more robust to preserve their core taste value, even if this means accepting a relatively limited yield increase. This provides key decision parameters for achieving true *“*cultivation according to variety and fertilization based on quality.

## 5 Conclusions

NAC varieties can achieve greater yield potential (mainly relying on increased panicle number) by increasing panicle fertilizer, but their appearance quality (e.g., chalkiness degree) is sensitive to high nitrogen levels. The processing quality (e.g., head rice rate) of low amylose varieties improves with increased panicle fertilizer, but their cooking/eating quality (e.g., taste value and pasting properties) deteriorates more significantly. The path to synergizing high yield and high quality varies by variety. For NAC varieties, panicle fertilizer management can be moderately aggressive to explore yield potential, but appearance quality must be monitored. For LAC varieties, a robust fertilization strategy should be adhered to, prioritizing the preservation of taste value and avoiding excessive nitrogen application.

## Data Availability

The raw data supporting the conclusions of this article will be made available by the authors, without undue reservation.

## References

[B1] Cao X. Sun H. Wang C. Ren X. Liu H. Zhang Z. (2018). Effects of late-stage nitrogen fertilizer application on the starch structure and cooking quality of rice. J. Sci. Food Agric. 98, 2332–2340. doi: 10.1002/jsfa.8723, PMID: 28991369

[B2] Chen H. Yang G. Xiao Y. Zhang G. Yang G. Wang X. . (2023). Effects of nitrogen and phosphorus fertilizer on the eating quality of indica rice with different amylose content. J. Food Composition Anal. 118, 105167. doi: 10.1016/j.jfca.2023.105167

[B3] GB/T 17891-2017 . (2017). High quality paddy. National Standard of the People’s Republic of China. (Beijing: Standardization Administration of the People’s Republic of China)

[B4] Guo C. Yuan X. Yan F. Xiang K. Wu Y. Zhang Q. . (2022). Nitrogen application rate affects the accumulation of carbohydrates in functional leaves and grains to improve grain filling and reduce the occurrence of chalkiness. Front. Plant Sci. 13, 921130. doi: 10.3389/fpls.2022.921130, PMID: 35812970 PMC9270005

[B5] Hu Y. Cong S. Zhang H. (2021). Comparison of the grain quality and starch physicochemical properties between japonica rice cultivars with different contents of amylose, as affected by nitrogen fertilization. Agriculture 11, 616. doi: 10.3390/agriculture11070616

[B6] Huang S. Zhao C. Zhu Z. Zhou L. Zheng Q. Wang C. (2020). Characterization of eating quality and starch properties of two Wx alleles japonica rice cultivars under different nitrogen treatments. J. Integr. Agric. 19, 988–998. doi: 10.1016/S2095-3119(19)62672-9

[B7] Jiang Y. Zhao C. Chen Y. Liu G. M. Zhao L. T. Liao P. Q. . (2023). Effects of nitrogen panicle fertilizer application on physicochemical properties and fine structure of japonica rice starch and its relationship with eating quality. Acta Agronomica Sin. 49, 200–210. doi: 10.3724/SP.J.1006.2023.12083

[B8] Jie H. S. (2004). Study on the Characteristics of starch and protein accumulation in grains of different amylose content rice varieties (Sichuan: Sichuan Agricultural University).

[B9] Liu K. Huang J. Zhou S. Q. Zhang W. Y. Zhang H. Gu J. F. . (2022). Effects of panicle nitrogen fertilizer rates on grain yield in super rice varieties with different panicle sizes and their mechanism. Acta Agronomica Sin. 48, 2028–2040. doi: 10.3724/SP.J.1006.2022.12068

[B10] Lu L. Hu X. Q. Chen M. X. Hu P. S. (2025). Chinese rice actually tastes ever more nice. Nature 34, 637. doi: 10.1038/d41586-024-04229-w, PMID: 39739095

[B11] Ma Z. Cao J. Chen X. Yu J. Guodong L. Xu F. . (2025a). Differences in carbon and nitrogen metabolism of soft japonica rice in southern China during grain filling stage under different light and nitrogen fertilizer conditions and their relationship with rice eating quality. Front. Plant Sci. 16, 1534625. doi: 10.3389/fpls.2025.1534625, PMID: 39935948 PMC11811539

[B12] Ma Z. Chen X. Cao J. Yu J. Zhu Y. Liu G. . (2025b). Carbon and nitrogen metabolism effects on eating quality in grains of diverse japonica rice cultivars from the middle and lower yangtze river. Food Energy Secur. 14, e70051. doi: 10.1002/fes3.70051

[B13] Ma Z. Yu J. Chen X. Cao J. Zhu Y. Liu G. . (2025c). Differences in starch and protein composition, morphological and structure, and their impacts on eating quality of soft japonica rice under different light and nitrogen fertilizer conditions in southern China. Food Chem. 474, 143204. doi: 10.1016/j.foodchem.2025.143204, PMID: 39921972

[B14] Shao Y. F. Zhu D. W. Zheng X. Mou R. X. Zhang L. P. Chen M. X. (2025). Development status and regional differences of japonica rice quality in the yangtze river delta region from 2002 to 2022. Chin. J. Rice Sci. 39, 264–276. doi: 10.16819/j.1001-7216.2025.240408

[B15] Shi S. Ma Y. Zhao D. Li L. Cao C. Jiang Y. (2023). The differences in metabolites, starch structure, and physicochemical properties of rice were related to the decrease in taste quality under high nitrogen fertilizer application. Int. J. Biol. Macromol 253, 126546. doi: 10.1016/j.ijbiomac.2023.126546, PMID: 37643670

[B16] Tao Y. Yao Y. Wang K. T. Xing Z. P. Zhai H. T. Feng Y. . (2022). Combined effects of panicle nitrogen fertilizer amount and shading during grain filling period on grain quality of conventional japonica rice. Acta Agronomica Sin. 48, 1730–1745. doi: 10.3724/SP.J.1006.2022.12039

[B17] Wang J. Zhang X. Xiao Y. Chen H. Wang X. Hu Y. (2024). Effect of nitrogen fertilizer on the quality traits of Indica rice with different amylose contents. J. Sci. Food Agric. 104, 8492–8499. doi: 10.1002/jsfa.13676, PMID: 38923540

[B18] Wang R. Z. Li T. Qian X. L. Zhang Y. Yang X. Z. Li G. Y. . (2025). Effects of nitrogen panicle fertilizer on yield, quality and aroma of southern japonica rice nanjing 9108. Scientia Agricultura Sin. 58, 2316–2332. doi: 10.3864/j.issn.0578-1752.2025.12.004

[B19] Wu Z. He L. Xiong Y. Chen K. Yang Z. Sun Y. . (2024). Effect of nitrogen fertilizer topdressing for panicle differentiation on grain filling of hybrid indica rice and its relationship with the activities of key enzymes for starch synthesis. Chin. J. Rice Sci. 38, 48–56. doi: 10.16819/j.1001-7216.2024.230401

[B20] Xi M. Wu W. Xu Y. Zhou Y. Chen G. Ji Y. . (2020). iTRAQ-based quantitative proteomic analysis reveals the metabolic pathways of grain chalkiness in response to nitrogen topdressing in rice. Plant Physiol. Biochem. 154, 622–635. doi: 10.1016/j.plaphy.2020.06.012, PMID: 32717594

[B21] Xia D. Wang Y. Shi Q. Wu B. Yu X. Zhang C. . (2022). Effects of wx genotype, nitrogen fertilization, and temperature on rice grain quality. Front. Plant Sci. 13, 901541. doi: 10.3389/fpls.2022.901541, PMID: 35937336 PMC9355397

[B22] Xiong R. Y. Tan X. M. Yang T. T. Pan X. H. Zeng Y. J. Huang S. . (2022). Relation of cooked rice texture to starch structure and physicochemical properties under different nitrogen managements. Carbohydr. Polymers 295, 119882. doi: 10.1016/j.carbpol.2022.119882, PMID: 35988987

[B23] Xiong R. Tan X. Yang T. Wang H. Pan X. Zeng Y. . (2023). Starch multiscale structure and physicochemical property alterations in high-quality indica rice quality and cooked rice texture under different nitrogen panicle fertilizer applications. Int. J. Biol. Macromol 252, 126455. doi: 10.1016/j.ijbiomac.2023.126455, PMID: 37633549

[B24] Yao S. Chen T. Zhao C. F. Zhou L. H. Zhao L. Liang W. H. . (2024). Analysis on appearance and cooking taste quality characteristics of different types of japonica rice in jianghuai rice-growing area. Chin. J. Rice Sci. 38, 709–718. doi: 10.16819/j.1001-7216.2024.240310

[B25] Zhang Q. Hu Y. J. Guo B. W. Zhang H. C. Xu X. J. Xu Y. F. . (2021). Study on the characteristics of soft japonica rice varieties with good taste and high yield in taihu lake area. Chin. J. Rice Sci. 35, 279–290. doi: 10.16819/j.1001-7216.2021.0907

[B26] Zhang Z. Chu G. Liu L. Wang Z. Wang X. Zhang H. . (2013). Mid-season nitrogen application strategies for rice varieties differing in panicle size. Field Crops Res. 150, 9–18. doi: 10.1016/j.fcr.2013.06.002

[B27] Zhao C. F. Yue H. L. Huang S. J. Zhou L. H. Zhao L. Zhang Y. D. . (2019). Eating quality and physicochemical properties in nanjing rice varieties. Scientia Agricultura Sin. 52, 909–920. doi: 10.3864/j.issn.0578-1752.2019.05.012

[B28] Zhou L. H. Ni X. H. Zhu Z. Chen T. Zhao Q. Y. Yao S. . (2023). Response of yield and rice quality to panicle fertilizer reduction of japonica Nanjing 3908 with good eating quality. Jiangsu Agric. Sci. 51, 63–67. doi: 10.15889/j.issn.1002-1302.2023.03.009

[B29] Zhou T. Zhou Q. Li E. Yuan L. Wang W. Zhang H. . (2020). Effects of nitrogen fertilizer on structure and physicochemical properties of ‘super’ rice starch. Carbohydr. Polymers 239, 116237. doi: 10.1016/j.carbpol.2020.116237, PMID: 32414446

[B30] Zhu D. Zhang H. Guo B. Xu K. Dai Q. Wei H. . (2017a). Effects of nitrogen level on yield and quality of japonica soft super rice. J. Integr. Agric. 16, 1018–1027. doi: 10.1016/S2095-3119(16)61577-0

[B31] Zhu D. Zhang H. Guo B. Xu K. Dai Q. Wei C. . (2017b). Effects of nitrogen level on structure and physicochemical properties of rice starch. Food Hydrocolloids 63, 525–532. doi: 10.1016/j.foodhyd.2016.09.042

