# Supplementary material for: Optimizing panicle fertilizer application based on amylose content for balancing yield and quality of japonica rice
Source: Front Plant Sci. 2026 Feb 17;17:1745001. doi: 10.3389/fpls.2026.1745001 (PMC12953504; doi:10.3389/fpls.2026.1745001)
Supplement: Supplementary file 1 [file Table1.docx]

**Appendix Table 1.** Significance of variance estimates related to years (Y), varieties (V), nitrogen level (N), and their interactions on rice yield and grain quality traits in NAC varieties and LAC varieties.

| Yield and quality traits | NAC | | |  | LAC | | |
| --- | --- | --- | --- | --- | --- | --- | --- |
|  | V | N | V×N |  | V | N | V×N |
| Panicle number | ns | * | ns |  | ns | ns | ns |
| Spikelets per panicle | ** | ns | ns |  | ** | ns | * |
| Total spikelets | * | * | ns |  | * | ns | ns |
| Seed setting rate | ns | ns | ns |  | * | ns | ns |
| Grain weight | ns | ns | * |  | ** | ns | ns |
| Yield | ns | ** | ns |  | ns | ns | ns |
| GL | ** | ns | ns |  | * | ns | ns |
| GW | * | ns | ns |  | ns | ns | ns |
| L/W | ** | ns | ns |  | ** | ns | ns |
| CR | ** | ns | ns |  | ** | ns | ns |
| CD | ** | ns | ns |  | ns | ns | ns |
| BR | * | ns | ns |  | ns | ns | ns |
| MR | ns | ns | ns |  | ns | ns | ns |
| HR | * | ns | ns |  | ** | ns | ns |
| Taste | ns | ** | ns |  | * | ns | ns |
| Protein content (%) | ns | ** | ns |  | ns | ** | ns |
| Amylose content (%) | ns | ns | ns |  | ns | ** | ns |
| Peak viscosity | ns | * | ns |  | ns | * | ns |
| Trough viscosity | ns | * | ns |  | ns | * | ns |
| Final viscosity | ns | * | ns |  | ns | ns | ns |
| Breakdown | ** | ns | ns |  | * | ns | * |
| Setback | ns | ns | ns |  | * | ns | ns |

Notes: NAC, japonica rice with normal amylose content; LAC, japonica rice with low amylose content.
